# Functional and histopathologic correlation in the fabry nephropathy with N215S genotype

**DOI:** 10.1186/s13023-025-03994-9

**Published:** 2025-09-02

**Authors:** Renzo Mignani, Gian Marco Berti, Gisella Vischini, Roberta Di Costanzo, Francesca Ciurli, Daniele Vetrano, Elena Biagini, Serena Serratore, Benedetta Fabbrizio, Gianandrea Pasquinelli, Gaetano La Manna, Irene Capelli

**Affiliations:** 1 Department of Medical and Surgical Sciences (DIMEC), Alma Mater Studiorum - University of Bologna, Bologna, Italy; 2 Nephrology, Dialysis and Kidney Transplant Unit, IRCCS Azienda Ospedaliero-Universitaria di Bologna, Bologna, Italy; 3 Cardiology Unit, Cardiac Thoracic and Vascular Department, IRCCS Azienda Ospedaliero-Universitaria di Bologna, Bologna, Italy; 4 Division of Cardiology, Cardiovascular Research Center, University Magna Graecia Catanzaro, Catanzaro, Italy; 5 Pathology Unit, IRCCS Azienda Ospedaliero-Universitaria di Bologna, Bologna, Italy

**Keywords:** Cardiovascular outcomes, Enzyme replacement therapy, Fabry disease, Late-onset, Renal outcome

## Abstract

**Rationale & objective:**

Late-onset Anderson-Fabry disease appears in adulthood, usually with prevalent cardiac involvement. The N215S (p.Asn215Ser) missense mutation represents the most frequent late-onset variant in European countries. The N215S nephropathy was then investigated from a clinical and histopathological point of view. Study design: Renal and cardiac assessments were evaluated at baseline. The standardized scoring system of histologic involvement proposed by International Study Group of Fabry Nephropathy was applied to kidney biopsies, performed before the treatment start. A deeper digital histological reinterpretation was provided in a subgroup. Treated patients’ renal function was evaluated at diagnosis (T0), after 5 years (T1) and after 10 years (T2) from the treatment start. Setting & Participants: 27 patients (11 males, 16 female) with a N215S variant were evaluated.

**Findings:**

Mean eGFR at diagnosis was 84.98 ± 26.4 mL/min/1.73m^2^, and 6 patients were CKD-stage 3–5. In the 24 treated patients, the mean T0 eGFR was 86.5 ± 28.01 mL/min/1.73m^2^; 26% with CKD-stage 3–5 (mean eGFR 45.3 ± 19.4 mL/min/1.73m^2^). At T1, the mean eGFR in 14/24 patients was 69.1 mL/min/1.73m^2^ and 35% with CKD-stage 3–5 (mean eGFR 33.3 ± 17.1 mL/min/1.73m^2^). T2 was evaluated in 6 patients, of which 5/6 (85%) with CKD-stage 3 (mean eGFR 43.2 ± 20 mL/min/1.73m^2^). 18/18 patients presented kidney biopsies with different degrees of podocyte vacuolization, while sclerosis was documented in 9/18. 14/18 patients showed a higher podocyte vacuolization score. Males showed significant greater interstitial fibrosis (p 0.016). Arteriolar intimal fibrosis significantly correlated with eGFR at baseline (p 0.09).

**Limitations:**

The main limitation was the number of patients, as well as the number of available kidney and cardiac biopsies.

**Conclusions:**

Even at early stages, N215S patients display typical histological abnormalities of Fabry disease. Moreover, chronic kidney disease seems to progress over time in treated patients, with arterial and arteriolar intimal fibrosis.

## Introduction

Fabry disease (FD) is an X-linked metabolic disorder caused by a genetic defect in the *GLA* gene located in the Xq22 region, which encodes the lysosomal enzyme α-galactosidase A (α-Gal A). Deficiency of this enzyme leads to progressive lysosomal accumulation of glycosphingolipids, such as gangliosides and ceramide-based molecules, that are present in multiple cell types, tissues and organs and contribute to tissue damage and clinical manifestations. Globotriaosylceramide (Gb3) and its deacetylated form, globotriaosylsphingosine (Lyso-Gb3), are the products primarily involved [1]. Clinically, FD may manifest with two main phenotypes. The classic early-onset form occurs prevalently in males, begins during childhood or adolescence with less than 1% α-Gal A enzyme activity, and is associated with a severe prognosis. It is characterized by cardiological and neurological features, such as acroparesthesias in the extremities, cerebrovascular events, left ventricular hypertrophy, and arrhythmias. Other characteristics can be angiokeratomas, sweating abnormalities, *cornea verticillata*, and proteinuria. The late-onset form has milder disease manifestations and a residual a-Gal activity that ranges from 2 to 20% of normal activity [2]. It usually occurs later in life (between the third and the sixth decade) and it is commonly associated with signs and symptoms of cardiac involvement. It is, therefore, typically referred to as an atypical variant. Indeed, a subject with a late-onset variant typically lacks early symptoms such as extremity pain and gastrointestinal complaints, and angiokeratoma or *cornea verticillata* are less common than in classic phenotypes. Cardiac involvement begins around the third/fourth life decade, and it is characterized by left ventricular hypertrophy, conduction disturbances, and arrhythmias. Disease severity in female patients may vary from almost asymptomatic to severely symptomatic, similarly to classic phenotypes [2].

More than 1000 different GLA mutations have been described in Fabry patients and the correlation of specific mutations with the clinical phenotype is far from straightforward [3]. High-throughput next generation sequencing (NGS)-based screening programs in high-risk populations and newborns have identified several novel GLA genotypes including some variants of unknown significance (VUS) not yet described or investigated [3]. Several mutations (mainly missense) have been reported to be associated with a later-onset phenotype of FD, including p.N215S, p.M296V, p.R301Q, and IVS4 + 919G > A [2, 4, 5, 6, 7, 8, 9]. Among these, the most common in Europe is p.N215S which may be earlier diagnosed as a result of neonatal, family or high-risk population screening initiative, as often occurs in hypertrophic cardiomyopathy patients (HCM) [10]. With concern of extracardiac manifestations, early reports on p.N215S variants describe patients with an overt cardiomyopathy without signs of renal involvement [5, 7, 11]. Subsequently, some studies have documented a limited prevalence of clinical signs of renal involvement between patients with a cardiac variant p.N215S genotype [10, 12, 13]. However, a scarcity of reports, generally single clinical case-based [14, 15], have documented the prevalence of histological lesions at diagnosis, and before the initiation of any therapy, from kidney biopsies in patients with this variant. Recently, a single centre case series described a large cohort of p.N215S patients that underwent a kidney biopsy. It revealed a widespread renal involvement, although it did not analyse the correlation between histological lesions and renal function, and the evolution of the latter over time [16]. It also lacked information regarding the baseline renal conditions and functional evolution of N215S patients over time.

The aim of this multicentre study was to evaluate the renal involvement in a wide cohort of patients with N215S variant from a clinical and histopathological point of view, with a prospective evaluation of renal involvement over time.

## Patients and methods

### Study design and patients

Twenty-seven genetically confirmed adult heterozygous (females) and hemizygous (males) FD patients with the p.N215S mutation were consecutively recruited between 2006 and 2023 in Bologna, Ferrara and Rimini University Hospitals. Patients were retrospectively analysed in an open cohort study. All patients with a p.N215S mutation within this study were either heterozygous (females) or hemizygous (males) carriers for the corresponding GLA mutations.

A comprehensive diagnostic work-up had been performed in all centres, including medical history and cardiac, renal, and neurologic evaluation. Almost all patients had started specific treatment with agalsidase alfa or beta and migalastat immediately after the diagnosis. Patients were evaluated at baseline (diagnosis/start treatment) (T0), after 5 years (T1) and after 10 years (T2) from the treatment start.

Renal assessments included urine analysis to assess the presence of proteinuria and the albumin/creatinine ratio (ACR) value from spot urine. Renal function was quantified by the estimated glomerular filtration rate (eGFR) using the Chronic Kidney Disease-Epidemiology Collaboration equation (CKD-EPI) [17].

Cardiac assessments included electrocardiography, echocardiography and cardiac magnetic resonance. Left ventricular hypertrophy (LVH) was defined as an end-diastolic maximal wall thickness (MWT), measured in short-axis, ≥ 12 mm. Cardiac magnetic resonance (CMR) was carried out on a 1.5 Tesla scanner using a standardized protocol. Native T1 mapping was performed in short axis slices using a modified Look-Locker inversion recovery sequence and measured in the basal/midseptum region of interest. Late gadolinium–enhanced (LGE) was performed 15 min after bolus injection of 0.1 mmol/kg gadobutrol (Gadovist).

Plasma Lyso-Gb3 levels were measured in the laboratory of the University of Rostock, in Germany. Reference values for Lyso-Gb3 were < 0.9 ng/ml in plasma, and for GLA activity > 32 nmol/h/mg protein in leukocytes. Enzyme replacement therapy (ERT)-naïve Lyso-Gb3 values were available for 10 males and 14 females.

Severe clinical events were evaluated as well. These included: (1) Major Adverse Cardiac Events (MACE): angina pectoris, arrhythmia, congestive heart failure myocardial infarction, significant cardiac procedure, like pace maker (PM) or implantable cardioverter-defibrillator (ICD) replacement; (2) Renal events: dialysis or renal transplantation; (3) Cerebrovascular events: haemorrhagic or ischemic strokes.

### Histological evaluation

Kidney biopsies were performed in accordance with current clinical practice (real-time ultrasound-guided percutaneous biopsies using an automated spring-loaded biopsy device). The procedures were straightforward, with few complications (post-biopsy bleeding, haematuria and minor perirenal hematomas). The standardized scoring system of histologic involvement in Fabry nephropathy proposed by International Study Group of Fabry Nephropathy (ISGFN) was applied to all kidney biopsy specimens [18]. Pathological findings were scored as present or absent (glomerular sclerosis, adhesions, peri-glomerular fibrosis, cellular GL3 inclusions) or classified/quantified as none (0, 0–9%), mild (1, 10–25%), moderate (2, 26–50%), severe (3, from 51% upwards) for tubulointerstitial fibrosis (IFTA), podocytes vacuoles of each individual glomerulus, arterial and arteriolar lesions.

Endomyocardial biopsies were taken from the right side of the interventricular septum, through right jugular vein, with a fluoroscopically directed bioptome. Endomyocardial fragments were treated according to standard protocols. Vacuolization was quantified as number of myocytes affected, expressed as percentage. Myocardial fibrosis was assessed on Azan Mallory trichome stained sections and classified as replacement or interstitial.

All the procedure were performed in the same centre by the same operator and the sample were examined by the same renal pathologist. A written informed consent, for enzymatic and molecular analysis, tissue biopsies and publication, was obtained by each patient participating to the study.

### Statistical analysis

Data were retrieved from the clinical records closest in time to each patient’s first consultation. Descriptive statistics were reported as mean ± standard deviation (SD) or as median and interquartile range (IQR) for continuous variables, according to their distribution. Categorical variables were presented as percentages (%).

Comparisons between groups were performed using the unpaired t-test or the Mann-Whitney U test for continuous variables, depending on their distribution. Categorical variables were compared using the Fisher’s exact test.

Associations between prespecified baseline variables — including histological, laboratory, and morphological parameters — and both baseline eGFR and annual eGFR decline were assessed using linear correlation analyses, applying Pearson’s or Spearman’s correlation coefficients (ρ) and corresponding *p*-values, as appropriate. A *p*-value < 0.05 was considered statistically significant. All analyses were conducted using R software (version 4.3.1).

## Results

This study evaluated the renal involvement of 27 patients (11 males and 16 females) with a N215S variant, belonging to 9 different families. Clinical characteristics at the diagnosis (baseline) are illustrated in Table 1. The mean age was 56 ± 19 years (61 ± 21 for males and 53 ± 17 for females). The mean age at diagnosis was 48 ± 17 years. Hypertension was observed in 6 patients (22.2%), with affected males having a mean age of 72.4 ± 10.9 years. ACE inhibitors or sartans were administered in 33.3% of patients.

**Table 1:** Clinical characteristics of N215S population at baseline. *GI: gastrointestinal; LVH: left ventricular hypertrophy; LGE: late gadolinium enhancement; CMR: cardiac magnetic resonance; PPM: permanent pacemaker; ICD: implantable cardioverter-defibrillator; MRI: magnetic resonance imaging; CKD: chronic kidney disease*,* eGFR calculated by CKD-EPI*

	Males [*n* = 11]	Females [*n* = 16]	Total [*n* = 27]
Age (years), *median (range)*	58.6 (18–86)	41.6 (25–85)	53.5 (18–86)
Acroparesthesia, *n (%)*	4/11 (36.4)	2/16 (12.5)	6/27 (22.2)
Angiokeratoma /anhidrosis, *n (%)*	0/11 (0.0)	0/16 (0.0)	0/27 (0.0)
Cornea verticillata, *n (%)*	0/11 (0.0)	0/16 (0.0)	0/27 (0.0)
GI symptoms, *n (%)*	1/11 (9.0)	1/7 ^a^ (14.3)	2/18 ^a^ (11.1)
Hearing impairement, *n (%)*	3/11 (27.3)	1/7 ^b^ (14.0)	4/18 ^b^ (22.2)
Hypertension, *n (%)*	4/11 (36.4)	2/16 (12.5)	6/27 (22.2)
LVH, *n (%)*	4/5 ^c^ (80.0)	3/10 ^d^ (30.0)	7/15 ^c, d^ (46.7)
LGE on CMR, *n (%)*	2/4 ^e^ (50.0)	1/9 ^f^ (11.1)	3/13 ^e, f^ (23.0)
PPM/ICD, *n (%)*	2/11 (18.2)	0/7 ^g^ (0.0)	2/18 ^g^ (11.1)
Stroke, *n (%)*	1/11 (9.0)	0/7 ^h^ (0.0)	1/18 ^h^ (5.6)
Brain MRI, *n (%)*	5/11 (45.5)	1/7 ^i^ (14.3)	6/18 ^i^ (33.3)
CKD stage 3, *n (%)*	4/11 (36.4)	2/16 (12.5)	6/27 (22.2)
Microalbuminuria, *n (%)*	11/11 (100.0)	16/16 (100.0)	27/27 (100.0)
Enzyme replacement therapy, *n (%)*	10/11 (90.9)	14/16 (87.5)	24/27 (88.9)
Migalastat, *n (%)*	0/11 (0.0)	0/16 (0.0)	0/27 (0.0)
Not treated, *n (%)*	1/11 (9.1)	2/16 (12.5)	3/27 (11.1)

^a^ Not evaluated in 9 patients,^b^ Not evaluated in 9 patients,^c^ Not evaluated in 6 patients

^d^ Not evaluated in 6 patients,^e^ Not evaluated in 7 patients,^f^ Not evaluated in 7 patients

^g^ Not evaluated in 9 patients,^h^ Not evaluated in 9 patients,^i^ Not evaluated in 9 patients

24/27 patients started treatment at diagnosis (T0), 17 patients with agalsidase alfa, 3 patients with agalsidase beta and 4 patients with migalastat. Only 3 patients did not yet started specific therapy. The only male was a young patient, with all investigations performed at diagnosis negative for organ damage. In particular, he did not present signs of cardiac involvement with a normal T1 mapping at CMR. During the follow-up, 4 patients were switched from agalsidase alfa to migalastat and 2 patients from agalsidase alfa to agalsidase beta. 14/24 patients (58%) reached the 5th year of treatment (T1) with agalsidase alfa (10 patients), beta (2 patients) and migalastat (2 patients); 6/24 patients (25%) reached the 10th year of therapy (T2) with agalsidase alfa (4 patients) and with agalsidase beta (2 patients).

### Functional aspects

The mean serum creatinine, in the cohort of the 27 recruited patients at diagnosis, was 1.02 ± 0.7 mg/dl, with eGFR of 84.98 ± 26.4 mL/min/1.73m^2^. At diagnosis, 6 patients presented already with an eGFR lower than 60 mL/min/1.73m^2^ (mean age 73.8 years) and 10 patients presented a pathologic proteinuria (637 ± 325 mg/24 hours). The mean serum Lyso-Gb3 at baseline was 2.6 ± 1.4 ng/ml (in 27 patients; 2.37 ± 1.45 ng/ml in females and 3.1 ± 1.22 ng/ml in males) (*n.v. < 1.9 ng/ml*). At echocardiogram, the median (IQR) MWT was 10 mm (9–16) and the left ventricular mass (LVMi) 87 g/m^2^ (66–176); 11 patients (44%) had LVH. Males showed higher MWT (19 mm (13–21) vs. 9.0 mm (8–10); p 0.008) and LVMi 177 g/m^2^ (137–320) vs. 81 g/m^2^ (64–102) (p 0.031) (Table 2).

**Table 2:**
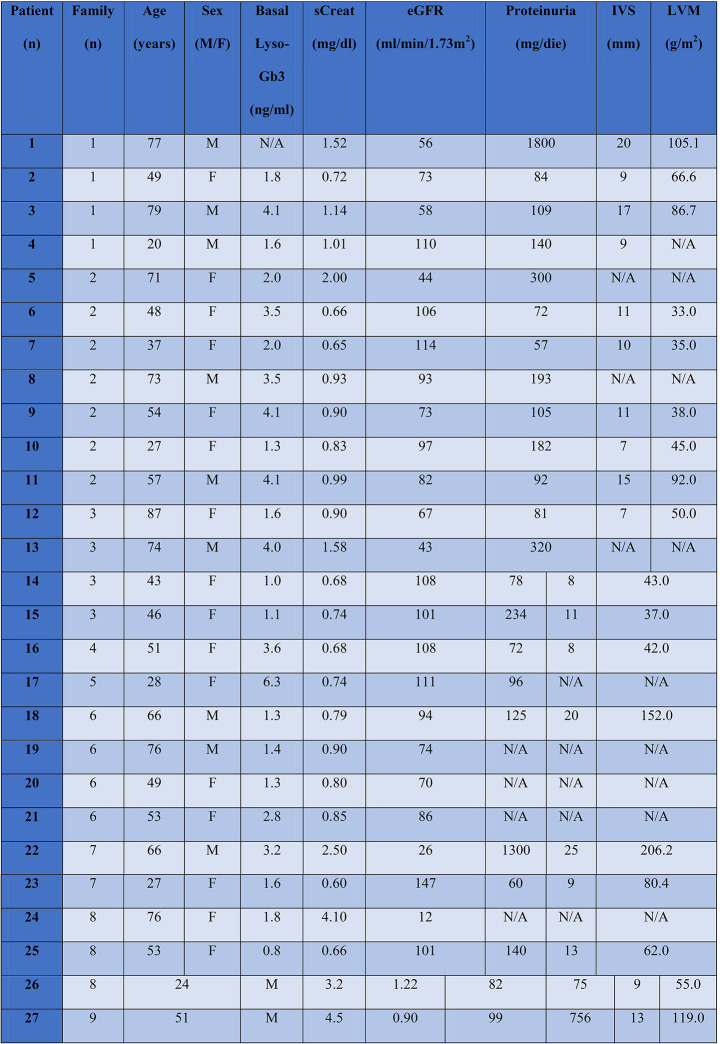
Functional characteristic of N215S population at baseline. *sCreat: serum creatinine; eGFR: estimated glomerular filtration rate; IVS: interventricular septum; LVM: left ventricular mass; M: males; F: females; Lyso-Gb3: globotriaosylsphingosine; N/A: not assessed*

**Fig. 1:**
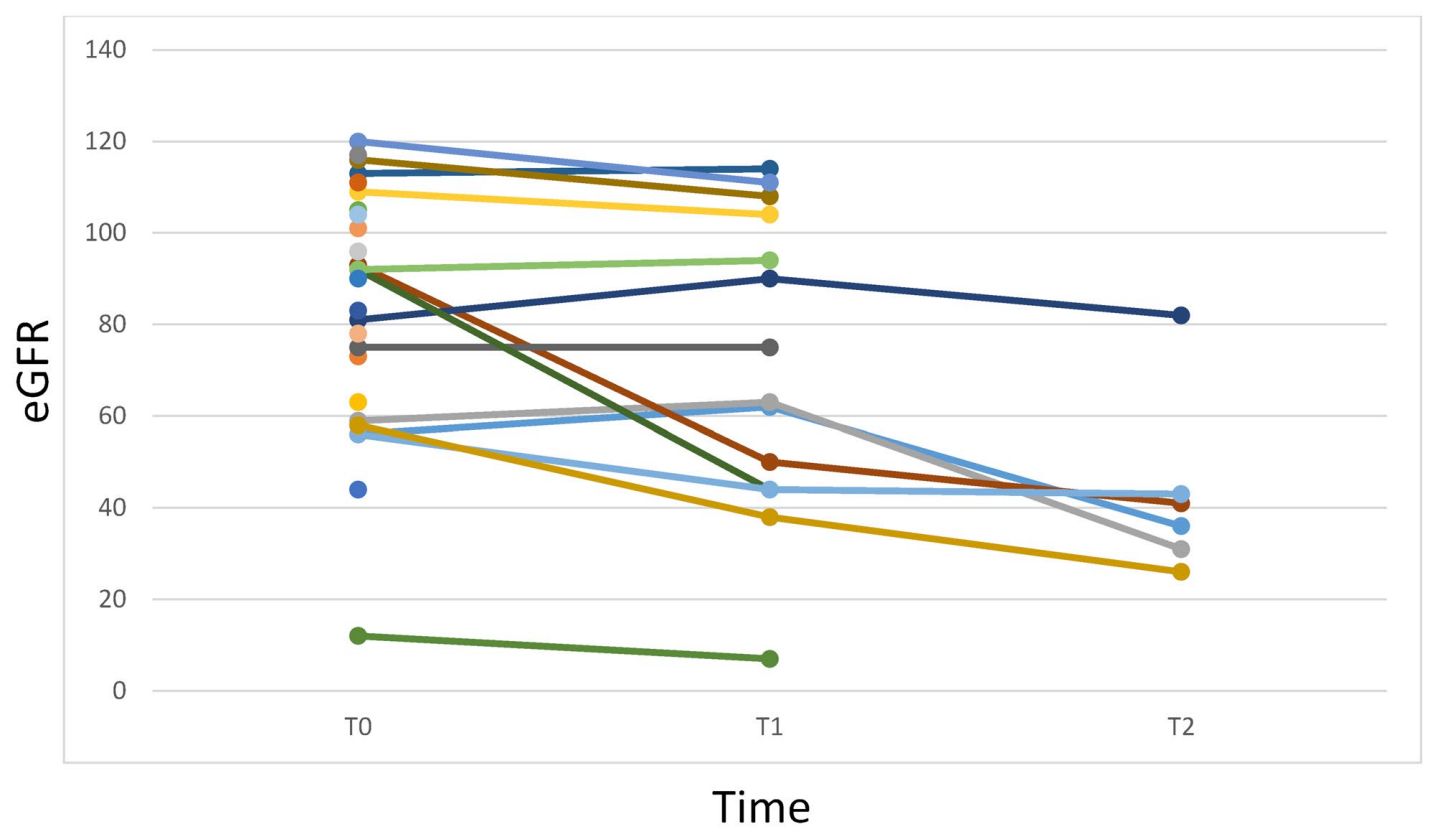
eGFR progression for patients. Renal function was evaluated at diagnosis (T0), after 5 years (T1) and after 10 years (T2) from the treatment start

24 patients were eligible for treatment with a mean eGFR at treatment start (T0) of 86.5 ± 28.01 mL/min/1.73m^2^ and a mean proteinuria of 531 ± 226 mg/24 hours. 5/24 patients presented a CKD-stage 3–5 (mean eGFR 45.3 ± 19.4 mL/min/1.73m^2^). At T1, the mean eGFR in 14/24 patients was 69.1 mL/min/1.73m^2^ and in 5/14 subjects was 33.3 ± 17.1 mL/min/1.73m^2^. Finally, in 5/6 patients that reached the 10th years of treatment (T2) a CKD-stage 3 was present (mean eGFR 43.2 ± 20 mL/min/1.73m^2^) (Fig. 1). 2/6 patients among those that presented an eGFR lower than 60 mL/min/1.73m^2^ were under treatment with ACE inhibitors or sartans and were affected by severe heart failure (both ICD carriers).

### Histological aspects

#### Renal biopsy

**Fig. 2:**
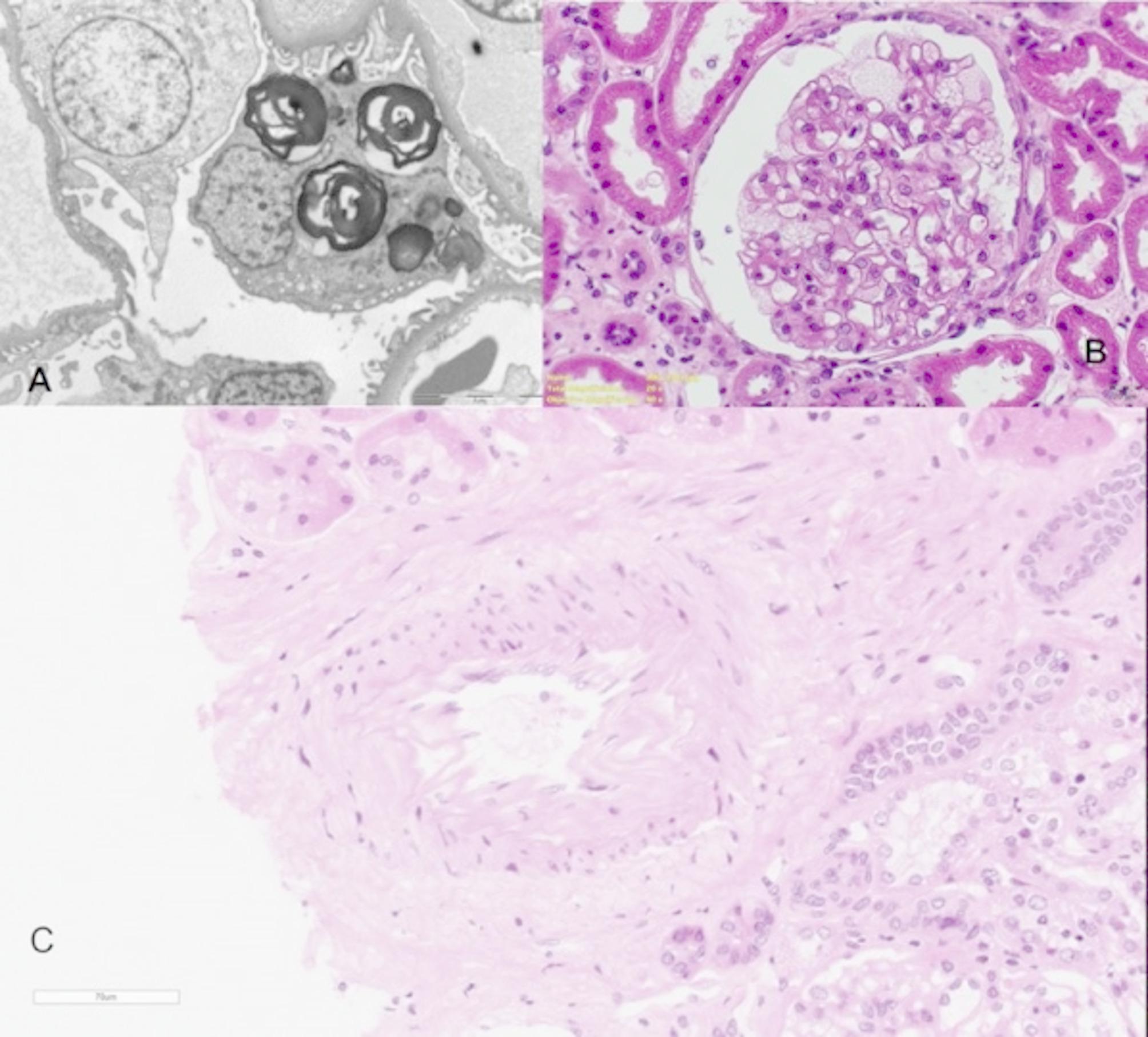
**(A)** Transmission Electron Microscopy. Distinctive Fabry inclusions are visible within the cytoplasm of an affected podocyte (right); in contrast, the podocyte on the left appears unaffected. Scale bar = 5 μm. **(B)** Light Microscopy, PAS staining. Most podocytes exhibit a “bubbly” appearance due to the accumulation of numerous lipid inclusions. Scale bar = 20 μm. **(C)** Light Microscopy, Hematoxylin & Eosin staining. An interlobular artery displaying distorted architecture with mild to moderate intimal fibrosis and a duplicated internal elastic lamina; no specific inclusions are observed. Scale bar = 70 μm

18/27 naïve-patients underwent a kidney biopsy at diagnosis. None of them manifested complications after the procedure. Light microscopy aspects of the kidney biopsies are illustrated in Table 3. The overall histological evaluation at light microscopy detected different degrees of podocyte vacuolization, global or segmental sclerosis (9/18 patients), and mild focal tubulo-interstitial fibrosis (13/18 patients) (Fig. 2). Arterial sclerosis was present in 44.4% of biopsies, mostly mild degree. Among this, only one patient was affected by hypertension. At the ultrastructural analysis, podocytes inclusions were present with several degrees of density. With the aim to provide a deeper histological characterization of N215S genotype, 14/18 patients (6 males and 8 females) underwent a digital reinterpretation of the biopsy, performed by an expert renal pathologist. In total, 237 glomeruli were analyzed by light microscopy, 47 of which were not scorable (fragment or damaged). The median glomeruli per biopsy were 12 (IQR = 11). Male patients showed statistically significant greater interstitial fibrosis (M 66.6% vs. F 28.6%, p 0.016) and nearly significant periglomerular capsular fibrosis (periglomerular capsular fibrosis Males 35%, Females 20%, overall 26%, p 0.070). Arteriolar intimal fibrosis significantly correlated with eGFR at baseline (ρ = -0.66, p 0.09). Moreover, eGFR slope significantly correlated with medial arterial hyalinosis (ρ = -0.63, p 0.01), IFTA (ρ = -0.68, p 0.006), and LV mass (ρ = -0.69, p 0.005) (Table 4).

**Table 3:** Summary of light microscopy data in renal biopsy specimens. Histological characteristics were evaluated using a quantitative or a semi-quantitative evaluation score (0 for 0–9%, 1 for 10–25%, 2 for 26–50%, 3 from 51% upwards). *N/A: not assessed; ISGFN: international study group of Fabry nephropathy*

Patient (*n*)	Glomeruli (*n*)	Nonsclerotic glomeruli (*n*)	Global Glomerulosclerosis	Segmental Sclerosis	Capsular Adhesion	Tubulo-Interstitial Fibrosis	Arterial sclerosis	Periglomerular fibrosis	Arteriolar Hyalinosis	Arterial Hyalinosis	Podocyte GL3 - ISGFN-score
1	36	35	1	0	0	0	0	0	0	0	0.20
2	15	15	0	0	N/A	0	0	N/A	0	0	0.96
3	8	8	0	1	N/A	1	1	N/A	1	N/A	0.43
4	13	13	0	0	N/A	0	1	N/A	0	N/A	1.86
5	18	18	0	0	N/A	0	0	N/A	0	N/A	0.06
6	12	10	2	0	N/A	1	0	N/A	0	N/A	1.08
7	5	5	0	0	N/A	1	1	N/A	0	N/A	N/A
8	7	5	2	0	N/A	1	2	N/A	2	N/A	1.00
9	17	17	0	0	0	0	0	0	0	0	N/A
10	9	9	0	0	0	1	0	0	0	0	N/A
11	22	21	1	0	N/A	1	0	N/A	1	N/A	1.00
12	26	26	0	0	N/A	0	0	N/A	0	N/A	1.00
13	27	26	1	0	N/A	1	1	N/A	1	N/A	0.83
14	20	20	0	1	N/A	1	1	N/A	1	N/A	1.15
15	16	15	1	0	N/A	0	1	N/A	0	N/A	1.47
16	14	13	1	0	N/A	1	1	N/A	0	N/A	1.88
17	0	0	0	0	N/A	2	N/A	N/A	3	N/A	N/A
18	7	7	0	0	0	0	N/A	0	0	0	0.00

**Table 4:** Correlation between baseline variables and annualized eGFR slope in the subgroup with biopsy digital reinterpretation. *ρ: correlation coefficient; eGFR: estimated glomerular filtration rate; IFTA: tubulointerstitial fibrosis; LV mass: left ventricular mass*

Characteristics	ρ	*p*-value
Medial arterial hyalinosis	-0.63	0.01
IFTA	-0.68	0.00
LV mass	-0.69	0.005
Age	-0.51	0.06

#### Endomyocardial biopsy

By histology (*n* = 8), all patients’ samples showed hypertrophied and vacuolated myocytes, with the median (IQR) percentage of vacuolated myocytes of 30% (18–58). Four (50%) had mild interstitial myocardial fibrosis. By ultrastructure analysis, all showed autophagolysosomes filled with fingerprint/zebra bodies osmiophilic lamellar inclusions.

## Discussion

The N215S variant represents the main late-onset phenotype variant of Fabry disease in European countries, while the IVS4 + 919G > A is the most frequent late-onset variant in the Asian population [13]. Up to now, this variant, mostly described in single cases or in small cohort studies, has been considered a cardiac variant due to the prevalent cardiac compromission compared to milder renal or central/peripheral nervous system involvement [12, 19]. Previous studies excluded the presence of proteinuria and renal function abnormalities in patients with N215S [7, 11]. More recently, few studies have described N215S cases with early renal involvement, as well as nephrotic syndrome and acute kidney injury due to minimal change disease (MCD) [15], or even cases of End Stage Renal Disease (ESRD) [20, 21]. However, all previous reports on N215S patients lacked in the description of histological features of this variant. Thus far, the largest study on N215S variant recruited 59 males and 66 females, with a mean age of 58 years. The incidence of chronic renal failure, as expression of renal involvement in this study, was very low, with only 17% of males and 5% of females with an eGFR < 60 mL/min/1.73m^2^ [13], although still higher than expected for the normal population in the same age range [22]. In a further retrospective single-centre observational study, 15 patients underwent a kidney biopsy at diagnosis, showing signs of Fabry nephropathy, such as vacuolization, podocytes hypertrophy and zebra bodies, even at early stages [16]. However, these studies did not point out any indication, neither on the renal function diagnosis or the evolution of the renal function over time.

In this study, we investigated the functional and histopathological status of renal and cardiac involvement of 27 patients with N215S variant at the moment of the diagnosis (baseline). To our knowledge, this is the first study on N215S Fabry genotype including clinical and histological characteristics combined with a functional assessment conducted over time. Therefore, using both clinical and histological data, this study valued significant findings which could help physicians in the daily clinical practice. At diagnosis, the clinical evaluation documented a normal renal function in almost every patient, while a CKD-stage 3–5 was already present in 6 patients. Moreover, the histological evaluation performed in 18/27 patients documented the presence of a Fabry nephropathy, even at early stages. Diffuse vacuolization, several degrees of sclerosis and the presence in all glomeruli of typical inclusions are the main proofs of an overt renal involvement at diagnosis.

Furthermore, from a clinical point of view, a functional deterioration was present in about 25% of patients at baseline. Besides, many patients manifested a renal progression even under therapies. Whereas the renal function seemed to maintain stability in almost all patients that reached the 5th year of treatment, it progressed to CKD-stage 3–5 in 85% of patients that reached the 10th year of treatment. However, all six patients presented a severe heart disease and the 50% of these were under treatment with ACE inhibitors or sartans.

Additionally, comparing the data obtained by our cohort with those published in the literature [18], which mainly concerned the classical variants, it was noted that, in patients comparable for functional stages, the Mean Vacuole Podocyte Score was significantly lower (0.92 ± 0.59 vs. 2.2 ± 0.8). This result may be due to the type of mutation itself which, as in all late-onset forms, allowed for residual enzymatic activity, unlike in untreated male patients with a classic phenotype, who exhibit the maximum score in PAS and semithin blue stain Sect. [18]. Interestingly, our cohort presented a higher mean podocyte vacuolization in females compared to males, even if not statistically significant, while interstitial and periglomerular capsular fibrosis were increased in males. This data differed from other published classic cohorts [18] and may suggests pathogenetic pathways involving the vascular and interstitial compartments. Concerning the correlation between histologic lesion and functional parameters, the vascular compartment was more informative. In this regard, as depicted in Table 5, supplementary analysis correlating histological features and clinical parameters were performed.

**Table 5:** Key differences in outcomes in the subgroup with biopsy digital reinterpretation. *eGFR: estimated glomerular filtration rate; MACE: major adverse cardiac events; F: females; M: males*

	F [*n* = 8]	M [*n* = 6]	Total [*n* = 14]	*p*-value
Δ eGFR, *median [IQR]*	-1.50 [-4.50, 3.00]	-10.50 [-14.25, -5.25]	-4.00 [-9.00, 1.25]	0.08
Δ eGFR%, *median [IQR]*	-1.40 [-3.89, 2.79]	-13.86 [-16.73, -6.20]	-3.84 [-10.70, 1.08]	0.033
MACE, *n (%)*	0 (0.0)	3 (50.0)	3 (21.4)	0.024
Slope, *median [IQR]*	-0.75 [-1.25, 0.00]	-3.95 [-4.88, -2.35]	-1.50 [-4.22, -0.37]	0.042
10% eGFR decline, *n (%)*	0 (0.0)	4 (66.7)	4 (28.6)	0.006

Male patients presented a worst renal (**Δ** eGFR, **Δ** eGFR %, eGFR slope, 10% eGFR decline) and cardiac (MACE) course, compared to female patients. Modifications in the vascular and interstitial compartments of N215S patients correlated significantly with the eGFR slope. Arteriosclerosis, pointed out by medial thickening of blood vessels (arterial and arteriolar), showed a notable correlation with proteinuria (p 0.028, p 0.050). Additionally, arterial, and arteriolar intimal fibrosis significantly correlated with eGFR at baseline (p 0.029, p 0.09). Although the presence of arteriosclerosis is more likely in older patients and, therefore, may not be pathognomonic of the disease, the absence of a history of hypertension in our cohort does not allow us to exclude a causal role of the genetic disorder.

Summarizing, the results of this study demonstrated that N215S patients presented typical Fabry disease histological abnormalities, even at early stages of the diagnosis. Moreover, the renal function, apparently stable in early stages, seemed to progress over time in treated patients. A worst Fabry nephropathy evolution seemed to correlate with a prevalent vascular and interstitial involvement. Therefore, an early and prompt histological evaluation of a kidney biopsy may help in framing the renal damage and guide clinicians as a predictive indication of the nephropathy progression. By contrast, interpreting a possible correlation between renal and cardiac histology is challenging due to the limited number of patients who underwent cardiac biopsy. Nevertheless, an interesting finding was the significant incidence of MACE in patients with the most significant decline in GFR.

Possible restrictions of this study are the retrospective design and the limited number of patients. Due to the multicentre approach, some parameters and DNA samples for X-chromosome inactivation analysis were not available for the entire study cohort. Other limitations are represented by limited data on Lyso-Gb3 values after the start of treatment. In this way, the lack of biopsies demonstrating the ultimately presence or absence of vacuolization corresponding to cellular Gb3 deposits, also represents a limitation. Finally, the limited number of cardiac biopsies makes it difficult to interpret a possible correlation between the two histological patterns of organ damage.

In conclusion, this study assessed the clinical and the histological features of a relatively large population with late-onset N215S variants, exploring the functional evolution after the start of treatment in the majority of patients. This study demonstrated that, at diagnosis, a widespread Fabry nephropathy is already present from both morphological and functional perspectives. The vascular and interstitial compartments of N215S kidney biopsies correlates significantly with eGFR slope, suggesting a possible role in the progression of the disease. For all these reasons we believe that the definition of this variant as “predominantly cardiac” should be reconsidered and abandoned, confirming the status of late-onset.

## Data Availability

Data sharing is not applicable to this article as no datasets were generated or analysed during the current study.
